# Myelinosome formation represents an early stage of oligodendrocyte damage in multiple sclerosis and its animal model

**DOI:** 10.1038/ncomms13275

**Published:** 2016-11-16

**Authors:** Elisa Romanelli, Doron Merkler, Aleksandra Mezydlo, Marie-Theres Weil, Martin S. Weber, Ivana Nikić, Stephanie Potz, Edgar Meinl, Florian E. H. Matznick, Mario Kreutzfeldt, Alexander Ghanem, Karl-Klaus Conzelmann, Imke Metz, Wolfgang Brück, Matthew Routh, Mikael Simons, Derron Bishop, Thomas Misgeld, Martin Kerschensteiner

**Affiliations:** 1 Institute of Clinical Neuroimmunology, University Hospital and Biomedical Center, Ludwig-Maximilians University Munich, 81377 Munich, Germany; 2 Department of Pathology and Immunology, University of Geneva, 1211 Geneva, Switzerland; 3 Division of Clinical Pathology, Geneva University Hospital, 1211 Geneva, Switzerland; 4 Max-Planck Institute of Experimental Medicine, 37075 Göttingen, Germany; 5 Department of Neurology, Georg-August University Göttingen, 37075 Göttingen, Germany; 6 Institute of Neuropathology, Georg-August University Göttingen, 37075 Göttingen, Germany; 7 Max von Pettenkofer-Institute and Gene Center, Ludwig-Maximilians University Munich, 81377 Munich, Germany; 8 Department of Physiology and Health Science, Ball State University, Muncie, Indiana 47306, USA; 9 German Center for Neurodegenerative Diseases (DZNE), 81377 Munich, Germany; 10 Institute of Neuronal Cell Biology, Technical University Munich, 80802 Munich, Germany; 11 Munich Cluster of Systems Neurology (SyNergy), 81377 Munich, Germany; 12 Department of Cellular and Integrative Physiology and Stark Neurosciences Research Institute, Indiana University School of Medicine, Indianapolis, Indiana 46202, USA; 13 Center of Integrated Protein Sciences (CIPS), 81377 Munich, Germany

## Abstract

Oligodendrocyte damage is a central event in the pathogenesis of the common neuroinflammatory condition, multiple sclerosis (MS). Where and how oligodendrocyte damage is initiated in MS is not completely understood. Here, we use a combination of light and electron microscopy techniques to provide a dynamic and highly resolved view of oligodendrocyte damage in neuroinflammatory lesions. We show that both in MS and in its animal model structural damage is initiated at the myelin sheaths and only later spreads to the oligodendrocyte cell body. Early myelin damage itself is characterized by the formation of local myelin out-foldings—‘myelinosomes'—, which are surrounded by phagocyte processes and promoted in their formation by anti-myelin antibodies and complement. The presence of myelinosomes in actively demyelinating MS lesions suggests that oligodendrocyte damage follows a similar pattern in the human disease, where targeting demyelination by therapeutic interventions remains a major open challenge.

## Abstract (web summary)

Oligodendrocyte damage is a key component of demyelinating diseases. Here, the authors use *in vivo* light and correlated electron microscopy in EAE mouse models, and find early damage occurs at the myelin sheath before spreading to the oligodendrocyte cell body.

## Graphical abstract

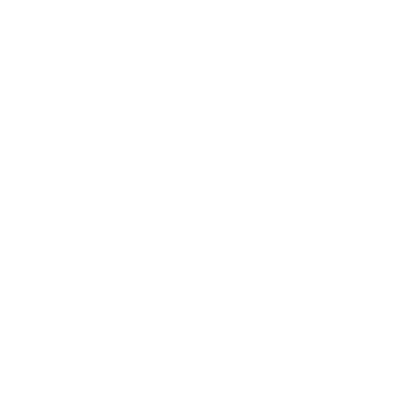

Although demyelination is a defining hallmark of multiple sclerosis (MS) and underlies the chronic impairment of axonal conduction in this disease, the cellular mechanisms of immune-mediated oligodendrocyte damage remain enigmatic^1^. For example, where neuroinflammatory damage is initiated along the extended geometry of an oligodendrocyte is unclear^2^, that is, whether at the cellular scale such damage spreads ‘centrifugally' (from the oligodendrocyte cell body to myelin^3^) or rather ‘centripetally' (from myelin to the soma^4^). Similarly, the earliest signs of demyelination in MS lesions are still unresolved. In principle, two different patterns could be posited: myelin damage could start at the myelin surface, where myelin is exposed to humoral and cellular immune mediators, or alternatively, pathology could first affect the myelin-axon adhesion, and hence the most distal extent of the oligodendrocyte. Furthermore, such myelin changes could be initiated focally at isolated sites on a myelin sheath or act segmentally affecting a larger stretch of myelin simultaneously, such as an entire internode. Disambiguating such possibilities is important to formulate clear molecular hypotheses about the mechanisms underlying oligodendrocyte damage and hence to build a rational foundation for future therapeutic approaches to prevent demyelination.

*In vivo* imaging has been a powerful analytical approach to resolve cell biological disease mechanisms in the intact nervous system. In the context of neuroinflammation, it has been applied to resolve the initiation of neuronal damage^5^,^6^, the subcellular correlate of axonal damage^7^, as well as at the immune cell dynamics that represent the proximate cause of these neuronal changes^8^. However, so far *in vivo* imaging has not been used to explore the cellular dynamics underlying neuroinflammatory oligodendrocyte damage.

Here, we take advantage of multi-spectral two-photon imaging of axons, oligodendrocytes and myelin in the intact spinal cord, viral labelling of single oligodendrocytes, correlated light and electron microscopy and immune modulation approaches, to show in mouse models of MS: (1) that the initial site of immune attack appears to be myelin rather than the oligodendrocyte soma and that damage then spreads centripetally, (2) that one of the early signs of demyelination is the focal formation of bulb-like structures, which we call myelinosomes, that appear on the surface of the myelin sheath and (3) that formation of such myelinosomes is promoted by anti-myelin antibodies and abrogated by complement depletion, compatible with a model of surface opsonization of myelin. Finally, this cellular mechanism of oligodendrocyte damage seems to be also operational in MS lesions, as we find the same sequence of oligodendrocyte damage, as well as evidence of myelinosome formation by high-resolution confocal imaging of actively demyelinating MS lesions.

## Results

### Demyelination precedes oligodendrocyte loss in an MS model

**Figure 1:**
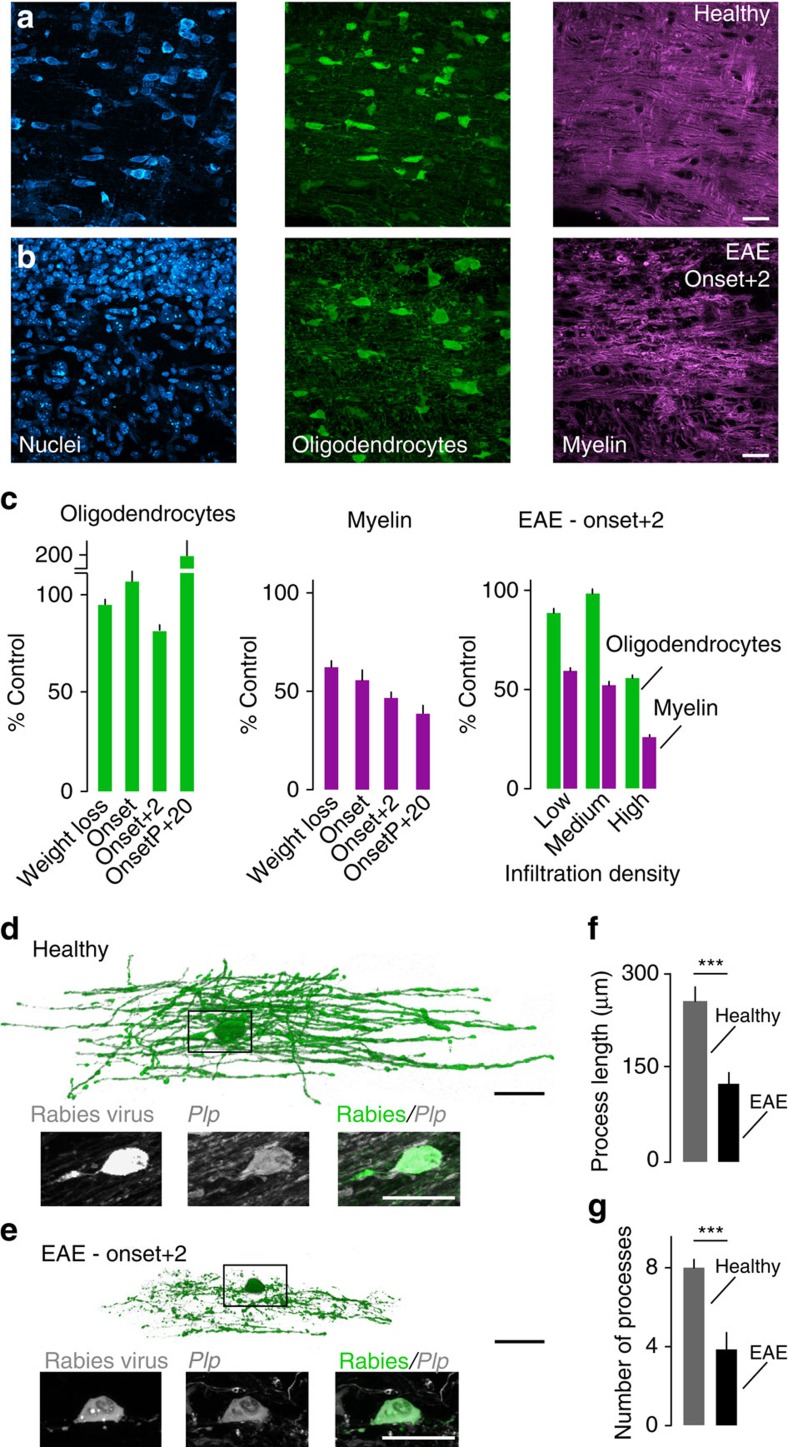
Centripetal spread of oligodendrocyte damage in EAE lesions. (**a**,**b**) Low magnification views of spinal cord sections of *PLP-GFP*/Biozzi ABH mice (oligodendrocytes labelled with GFP, green; nuclei with NT, cyan; myelin with MBP, magenta). In EAE (**b**) compared with healthy controls (**a**) invading immune cells and myelin disruption are apparent. (**c**) Quantification of oligodendrocyte number (green bars) and myelin length (magenta bars) at different time-points (Weight loss, *n*=3 mice; Onset, *n*=3 mice; Onset+2, *n*=6 mice; Onset of progression+20 days, OnsetP+20, *n*=3 mice) and related to infiltration density in lesions analyzed at Onset+2 days (*n*=6 mice). All values are shown as percentage of control (*n*=3 mice). (**d**–**g**) Three-dimensional renderings of confocal image stacks of single oligodendrocytes infected with rabies virus in *PLP-GFP*/Biozzi ABH mice, revealing oligodendrocyte process damage in EAE (**e**) compared with healthy controls (**d**) that affects both the length (**f**) and number of processes (**g**; *n*=8 oligodendrocytes for each group). Insets below in **d**,**e** show the respective oligodendrocyte somata. (**a**,**b**) Scale bar, 25 μm; (**d**,**e**) Scale bar, 100 μm (rendering) and 20 μm (inset). ***P<0.001 (**f**, *P*=0.0001; **g**, *P*=0.0007; *t*-test).

To understand where along the oligodendrocyte's extent damage is initiated during neuroinflammation, we first quantified distinct aspects of oligodendrocyte pathology in an animal model of MS, experimental autoimmune encephalomyelitis (EAE). For this purpose, we induced EAE in Biozzi ABH mice by active immunization with the myelin oligodendrocyte glycoprotein (MOG). In this model, which recreates key histopathological features of MS, the mice initially develop relapses, which later give way to a steady progression of the disease^9^. To reliably visualize oligodendrocyte cell bodies, we crossed Biozzi ABH with PLP-GFP/BL6 mice, in which the expression of GFP is driven by the PLP promoter, thus allowing the visualization of oligodendrocytes and their processes^10^. Experiments were performed with F1 hybrids, in which the clinical course and histopathological appearance of the disease is similar to wild-type Biozzi ABH mice (Supplementary Fig. 1). We perfused mice at different time-points during the course of EAE (at weight loss, at the onset of clinical symptoms, two days after the onset and at a chronic time-point, 20 days after onset of the progressive disease phase) and used confocal microscopy to determine the number of oligodendrocytes, as well as myelin density (based on MBP staining) in relation to the local infiltration density (based on Neurotrace staining). Our analysis showed that the proportion of lost myelin exceeded the percentage of oligodendrocyte loss throughout the course of EAE. For example, already nearly half of the myelin was lost in lesions analyzed during the initial stages of the disease, while oligodendrocyte numbers were unchanged at this time. Likewise, at the peak of disease activity (2 days after onset of clinical symptoms) myelin loss appeared to be the predominant alteration in all areas of a neuroinflammatory lesion (Fig. 1a–c): In the lesion centre (defined by a high density of infiltrating cells) myelin was largely lost, while more than half of the oligodendrocyte cell bodies were still present. Likewise, in lesion areas with only a moderate or low number of infiltrating immune cells, we observed a substantial decrease of myelin density in the absence of measureable oligodendrocyte loss. To confirm at the single-cell level that the spread of oligodendrocyte damage indeed follows a centripetal pattern, we established a low-cellular density labelling approach based on the injection of a diluted mCherry-expressing rabies virus^11^. When injected into the lumbar dorsal columns of *PLP-GFP*/Biozzi ABH spinal cords this technique allowed us to reconstruct single oligodendrocytes and their processes using confocal microscopy. Quantitative analysis revealed that oligodendrocytes in spinal EAE lesions preserved shorter and fewer processes than oligodendrocytes in healthy mice (Fig. 1d–g). In line with these observations in fixed tissue, *in vivo* two-photon imaging of single-labelled oligodendrocytes at different stages of the neuroinflammatory lesion revealed that progressively shorter and less oligodendrocyte processes were observed, confirming the centripetal progression of oligodendrocyte pathology (Supplementary Fig. 2). During time-lapse recordings of several hours, almost no changes were observed, suggesting that oligodendrocytes stably persisted for longer periods of time despite extensive damage to their processes. Moreover, this ‘amputated' state appears not to be an immediate step towards cell loss, as during our *in vivo* imaging experiments, we did not see any cell die (total of 100 h of imaging of 20 oligodendrocytes in 18 mice).

Taken together, these results point to a sequence of damage that starts with myelin loss followed by degeneration of oligodendrocyte processes and only in some cases, cell death. One conclusion from these results is that to understand how neuroinflammatory oligodendrocyte damage is initiated, we have to focus on the myelin sheaths themselves.

### *In vivo* imaging reveals formation of myelinosomes

**Figure 2:**
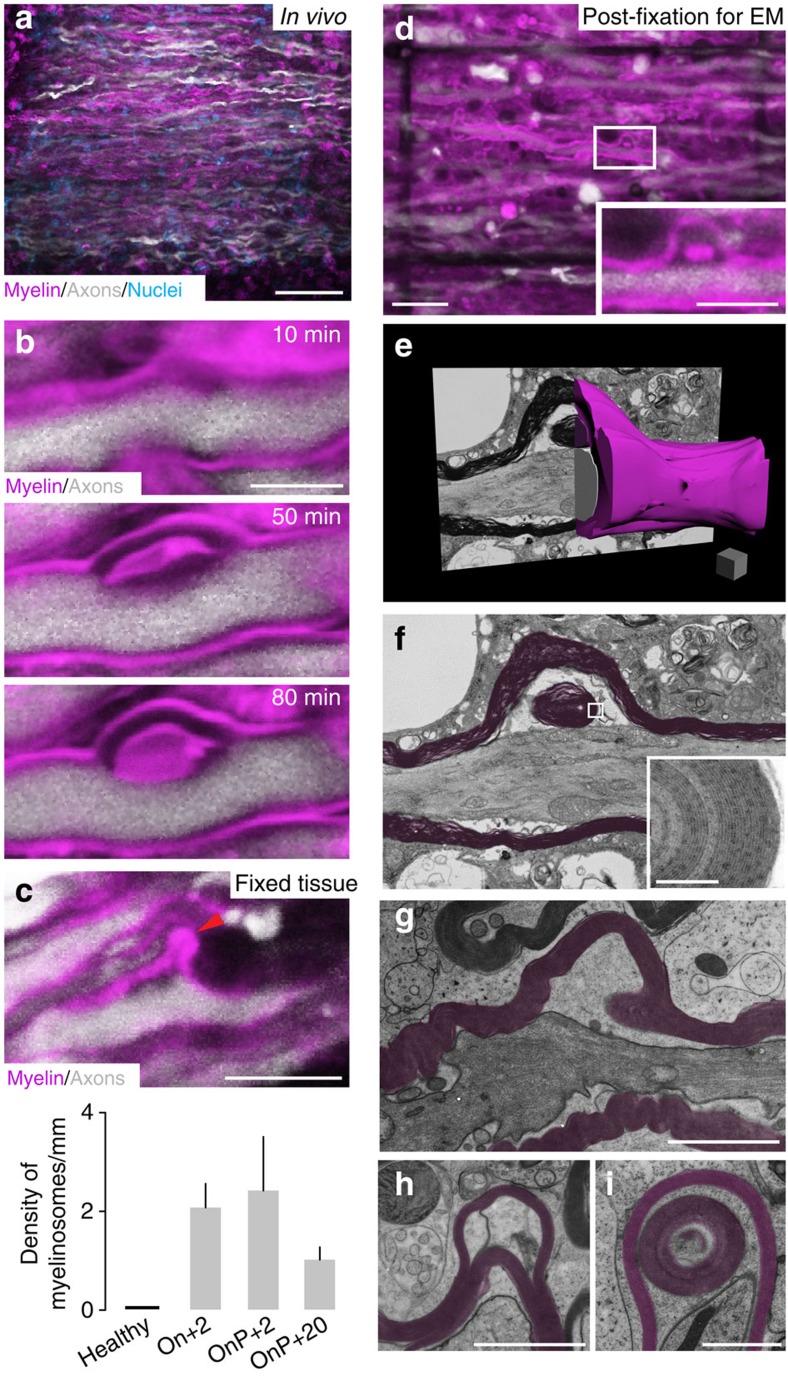
*In vivo* imaging and correlated electron microscopy reveal myelinosome formation in acute neuroinflammatory lesions. (**a**) *In vivo* image of an acute EAE lesion in a *Thy1-YFP16*/Biozzi ABH mouse revealing swollen axons and disrupted myelin sheaths in areas of cell infiltration (axons, white; nuclei, cyan; myelin, magenta throughout the figure). (**b**) *In vivo* time-lapse showing the formation of a myelin out-folding (‘myelinosome') along an EAE axon. (**c**) Confocal image of a myelinosome stained for myelin (MBP) in a *Thy1-YFP16*/Biozzi ABH mouse perfused at 2 days after EAE onset. The graph shows the density of myelinosomes per mm axon at different time-points of EAE (healthy control, *n*=3 mice, 254 axons segments with 6.27 mm of total axon length were evaluated; Onset+2, *n*=6 mice, 449 axon segments with 15.8 mm of total axon length were evaluated; Onset of Progression+2, OnP+2, *n*=3 mice, 238 axon segments with 9.1 mm of total axon length were evaluated; Onset of progression+20, OnP+20, *n*=5 mice, 480 axon segments with 16.6 mm of total axon length were evaluated). (**d**) ‘Near infrared branding' marks (dark lines) allow correlated ultrastructural reconstructions of myelinosomes (the boxed example is reconstructed in **e** and magnified in inset). (**e**) Three-dimensional rendering of the myelinosome shown in **d**. (**f**) Single ultrathin section through the centre of the myelinosome, showing the detachment of myelin (pseudocoloured magenta) from the axon, and inclusion of a myelin sphere inside (inset). (**g**–**i**) Electron micrographs from high-pressure frozen spinal EAE tissue illustrating the presence of myelinosomes that encompass all myelin leaflets (**g**), that show splitting of the myelin sheaths (**h**) and that contain internal myelin structures (**i**). (**a**) Scale bar, 50 μm; (**b**,**c**) Scale bar, 5 μm; (**d**) Scale bar, 20 μm (overview) and 10 μm (inset); (**e**) Scale bar, 1 μm (scale cube); (**f** (inset)) Scale bar, 200 nm; (**g**,**h**) Scale bar, 2 μm; (**i**) Scale bar, 1 μm.

To directly visualize early signs of neuroinflammatory myelin damage, we induced EAE in *Thy1-YFP16*/Biozzi ABH mice, in which many spinal axons are marked by YFP^12^, and labelled myelin by local application of MitoTracker Red (which acts as a lipophilic myelin dye at the concentration used^13^). We then imaged regions of active inflammation (identified based on the accumulation of immune cells labelled with the vital dye Popo-1-Iodide^13^) in the superficial dorsal spinal cord by *in vivo* two-photon microscopy (Fig. 2a). In the resulting time-lapse movies, we could in some cases (*n*=13) observe the *de novo* formation of bulb-like myelin structures, which we refer to as ‘myelinosomes' (adopting a nomenclature used for fragments shed by other cells^14^). These myelinosomes appeared at discrete spots along the internode and gradually lifted off the parent axons, in the process sometimes forming a myelin dye-labelled inclusion sandwiched between the myelin out-folding and the axon (Fig. 2b; Supplementary Movie 1). Subsequent confocal microscopy analysis of fixed EAE tissue stained with an anti-MBP antibody confirmed the presence of myelinosomes in neuroinflammatory lesions independently of *in vivo* imaging and vital dye application. A particularly high density of myelinosomes was present during active inflammation 2 days after EAE onset, as well as at 2 days following the onset of EAE progression. Myelinosomes were still present, albeit at a lower density, during the chronic phase of the disease (Fig. 2c). Further analysis revealed that myelinsomes are not only present in the Biozzi EAE model, but are also observed in *Thy1-YFP16* mice on a C57BL/6 background (*Thy1-YFP16*/BL6) immunized with recombinant rodent or human MOG (EAE induced by rodent MOG in *Thy1-YFP16*/BL6 mice: 1±0.1 myelinosomes/mm axon at Onset+2 days; *n*=5 mice, 252 axon segments with 9 mm of total axon length were evaluated; EAE induced by human MOG in *Thy1-YFP16*/BL6 mice: 1.5±0.6 myelinosomes per mm axon at Onset+2 days; *n*=3 mice, 286 axon segments with 6.9 mm total axon length were evaluated). In contrast, such structures were observed neither in healthy control animals (Fig. 2c), nor in a centrifugal model of oligodendrocyte pathology induced by the injection of ethidium bromide into the lumbar spinal cord (0±0 myelinosomes; *n*=3 healthy mice, 217 axon segments, 5 mm total axon length; likewise 0±0 myelinosomes were observed in 3 saline-injected mice, 183 axon segments, 4.1 mm total axon length; Supplementary Fig. 3).

To understand the ultrastructure of myelinosomes, we used the near infrared branding (NIRB) technique^15^ to reconstruct myelinosomes identified under the light microscope by serial sectioning transmission electron microscopy (Fig. 2d–f). The ultrastructural analysis confirmed that myelinosomes, as well as the myelin dye-positive inclusions, are composed of structures that showed the membrane layering characteristic of compacted myelin. To better visualize internal myelin ultrastructure, we analyzed high-pressure frozen samples of neuroinflammatory lesions induced in Biozzi ABH mice. This analysis revealed two morphological features that subcategorize myelinosomes: (i) myelin outfolding could either result from the detachment of the entire myelin sheath from the axon surface or a splitting of the myelin sheets, resulting in myelinosomes comprised of only some superficial myelin leaflets (typically including about 50% of the myelin thickness); (ii) some myelinosomes contained myelin inclusions inserted either between the axon and the detached myelin or between flared myelin sheaths (Fig. 2g–i; Supplementary Fig. 4). Such ultrastructural myelin changes appear to be specific for immune-mediated oligodendrocyte damage, as they are essentially absent in a model of toxic demyelination induced by the copper chelator cuprizone (one structure resembling a myelinosome was observed in 181 myelin profiles in the corpus callosum from 6 mice, half of which were examined at 2 or 3 weeks of cuprizone treatment, respectively, versus no myelinosomes in 172 myelin profiles in the corpus callosum of 3 control mice; cf. 16 myelinosomes that were observed in 115 myelin profiles in the spinal cord from 3 EAE mice versus none in 125 myelin profiles in the spinal cord of 3 control mice).

### Antibody–complement complexes promote myelinosome formation

**Figure 3:**
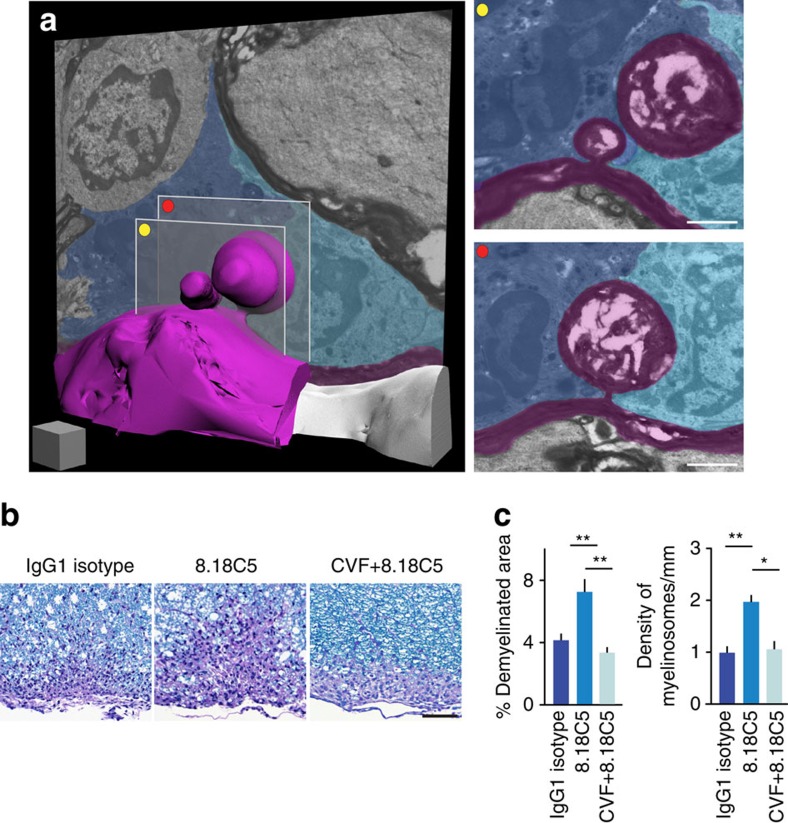
Myelinosome formation is mediated by antibody opsonization and macrophage engulfment. (**a**) Three-dimensional electron microscopy reconstructions reveal that myelinosomes (here examples composed of a few myelin leaflets; pseudocoloured in magenta) are surrounded by cells that show the ultrastructural hallmarks of phagocytes (pseudocoloured in cyan and blue). Single electron microscopy planes of the myelinosomes are shown on the right. (**b**,**c**) Anti-myelin antibodies and complement initiate demyelination and myelinosome formation as revealed by LFB/PAS stainings (**b**; myelin, blue) to assess the area of demyelination and confocal microscopy analysis of MBP-immunostained sections to quantify myelinosome density, both performed in EAE animals (*Thy1-YFP16*/BL6) treated with anti-MOG antibody (8.18C5) without or with complement depletion (cobra venom factor, CVF; for the area of demyelination were analyzed: *n*=11 mice injected with IgG1 isotype control antibody, *n*=11 mice treated with 8.18C5 and *n*=4 mice receiving CVF+8.18C5; for the quantification of density of myelinosomes were analyzed: *n*=14 mice injected with IgG1 isotype control antibody, *n*=15 mice treated with 8.18C5 and *n*=8 mice receiving CVF+8.18C5). (**a**) Scale bar, 1 μm (cube), and 1 μm (insets); (**b**) Scale bar, 50 μm. *P<0.05; **P<0.01 (for analysis of demyelinated area: Kruskal–Wallis followed by Dunn's multiple comparison test; for analysis of myelinosomes formation: one-way ANOVA followed by Bonferroni's multiple comparison tests).

To identify potential immune mediators that can induce myelinosome formation, we first investigated the cellular environment of myelinosomes in electron microscopy reconstructions. We found that in our samples, myelinosomes were often in the immediate vicinity of phagocytes, which in some cases engulfed the myelinosomes (Fig. 3a; Supplementary Movie 2). To better characterize the phagocyte population surrounding sites of myelinosome formation, we induced EAE in *CCR2*^*RFP/+*^*/*Biozzi ABH mice. In this knock-in mouse strain monocyte-derived macrophages, the primary phagocyte population associated with demyelination in EAE^16^, are labelled with RFP. We perfused mice at the peak of disease activity (2 days after onset) and counterstained the tissue for MBP to detect myelinosomes. To probe whether macrophages are more likely to interact with myelinosomes than morphologically unaffected myelin, we compared the probability of a macrophage to be in close apposition (<1 μm distance) to a myelinosome to the probability of a phagocyte contact on the opposite side of the axon (where due to the low density of myelinosomes, such a structure is typically absent). Our confocal analysis showed that 83.7% of the myelinosomes examined (*n*=43 myelinosomes from four mice) were in close apposition with blood-derived macrophages (RFP^+^ cells), while only 34.9% of the (unaltered) myelin on the other side showed such appositions. To further assess the potential contribution of tissue-resident microglia cells to myelinosome formation, we counterstained additional tissue sections with the phagocyte marker Iba1. Here, none of the myelinosomes examined (*n*=15 myelinosomes from three mice) were in contact with microglial cells (Iba1^+^/RFP^−^ cells), while in 20% of the cases microglia cells were found in close apposition with the corresponding unaffected side of the axon. Taken together, these analyses suggest a preferential association of myelinosomes and blood-derived macrophages.

One candidate mechanism that could initiate such a macrophage-mediated immune attack against myelin is the humoral immune response. To directly test whether anti-myelin antibodies can induce myelinosome formation, we induced EAE in *Thy1-YFP16*/BL6 mice by immunization with MOG_35–55_ peptide. In this variant of EAE, B cells and antibodies play only a minor role and primary demyelination is limited^17^. Demyelination can however be substantially enhanced by the transfer of the MOG-reactive monoclonal antibody, 8.18C5 (ref. 18). Indeed, in this EAE model, we found a comparably low density of myelinosomes, while anti-MOG antibody transfer not only increased the area of demyelination, but also the local density of myelinosomes within the demyelinated area (Fig. 3b,c). At the same sites, T cells and macrophage densities were not significantly altered (Supplementary Fig. 5). These experiments indicate that anti-myelin antibodies can induce the formation of myelinosomes in a neuroinflammatory environment, and raise the question how such antibodies prime phagocyte activation. One possible intermediate of such phagocyte priming could be the activation of complement components by myelin-bound antibodies^19^. To probe the involvement of this mechanism in myelinosome formation, we treated *Thy1-YFP16*/BL6 mice with Cobra Venom Factor (CVF) to deplete complement^20^ and in parallel induced myelinosome formation by transfer of the 8.18C5 antibody. Complement depletion prevented antibody-induced myelinosome formation in areas of demyelination (Fig. 3b,c), again leaving infiltration densities unaffected (Supplementary Fig. 5). In line with previous work^18^, complement depletion also reduced the overall size of the demyelinated area. Together these results indicate that antibody–complement complexes play an important role in the initiation of myelinosome formation and subsequent myelin loss in neuroinflammatory lesions.

### Myelinosomes are present in actively demyelinating MS lesions

**Figure 4:**
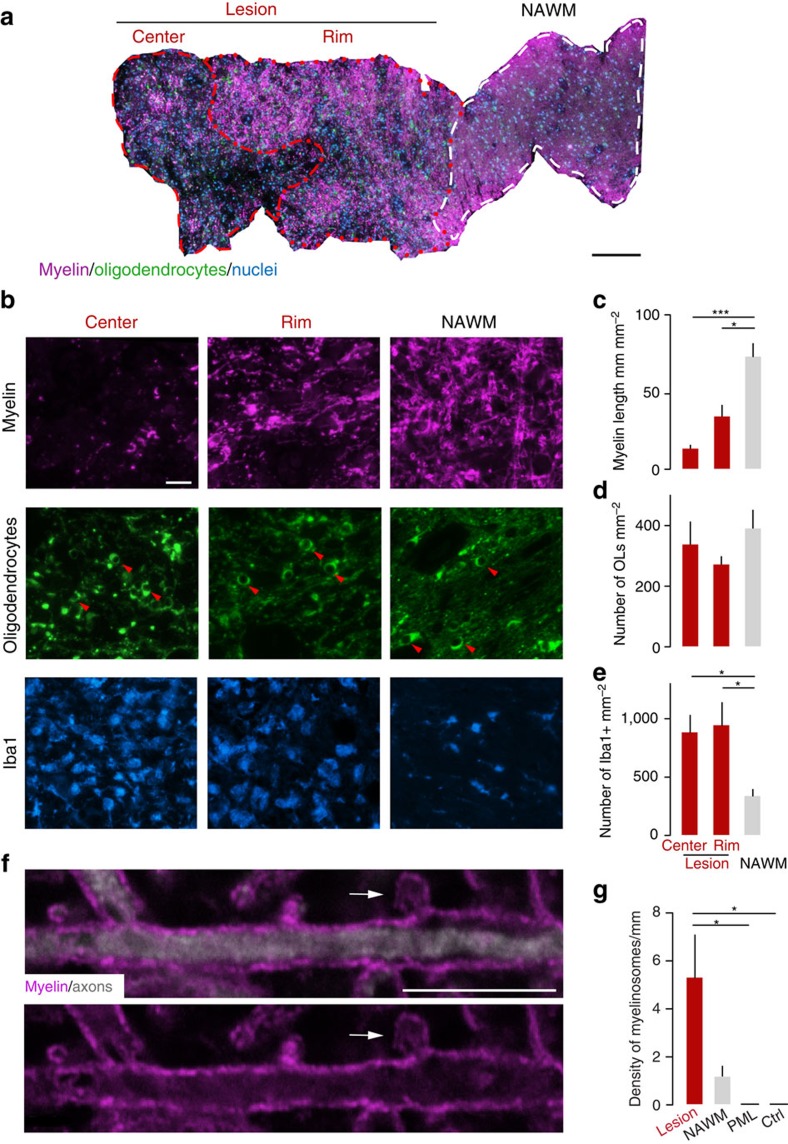
Oligodendrocyte pathology in actively demyelinating MS lesions spreads centripetally and involves myelinosome formation. (**a**) Overview of a biopsy containing an actively demyelinating MS lesion stained for oligodendrocytes (NogoA, green), myelin (MBP, magenta) and nuclei (DAPI, cyan) that is segmented in three different areas, the lesion centre, lesion rim and normal-appearing white matter (‘NAWM'). (**b**) Higher magnification views from areas as defined in **a** (also stained for Iba1 to reveal the activation of mononuclear phagocytes, cyan). Arrowheads, oligodendrocyte somata. (**c**–**e**) Quantification shows loss of myelin (**c**; lesion centre, *n*=25 areas from 11 biopsies; rim of the lesion, *n*=15 areas from 11 biopsies and NAWM, *n*=10 areas from 4 biopsies), while oligodendrocyte density is preserved (**d**; lesion centre, *n*=23 areas from 11 biopsies; rim of the lesion, *n*=29 areas from 11 biopsies and NAWM, *n*=16 areas from 7 biopsies) in areas of phagocyte infiltration (**e**; lesion centre, *n*=5 areas from 3 biopsies; rim of the lesion, *n*=6 areas from 3 biopsies and NAWM, *n*=5 areas from 3 biopsies). (**f**) High-power view of a myelinosome (arrow; MBP, magenta; neurofilament, white) in an actively demyelinating MS lesion. (**g**) A comparably high density of myelinosomes is present in MS lesions, while only few are found in normal-appearing white matter in MS and none are detected in cases of PML or in controls without inflammatory CNS disease (‘ctrl', *n*=3 autopsy cases; NAWM, *n*=7 biopsies; MS lesion, *n*=7 biopsies; PML, *n*=4 autopsy cases). (**a**) Scale bar, 200 μm; (**b**) Scale bar, 20 μm; (**f**) Scale bar, 10 μm. *P<0.05; **P<0.01 (Kruskal–Wallis followed by Dunn's multiple comparison tests).

To investigate whether myelinosome formation is also an early step of oligodendrocyte damage in the human disease, we analyzed actively demyelinating lesions in brain biopsies from MS patients (Supplementary Table 1). To determine, whether in MS oligodendrocyte damage followed a ‘centripetal' pattern, we used the same approach as in EAE (cf. Fig. 1) and performed triple immuno-labelling to reveal oligodendrocytes (stained for NogoA), myelin (MBP) and microglia/macrophages (Iba1) on sections counterstained with the nuclear dye DAPI (Fig. 4a; Supplementary Fig. 6). Quantitative analysis of early active lesions^21^ showed that myelin was almost completely lost in the centre and substantially reduced at the rim region of the lesion, while it was preserved in the normal-appearing white matter (NAWM). In contrast, the density of NogoA-positive oligodendrocytes was comparable between the lesion areas and the adjacent NAWM (Fig. 4b–e). These results are consistent with a centripetal spread of damage in oligodendrocytes located in active MS lesions, that is damage that primarily affects myelin integrity, and only to a lesser extent oligodendrocyte survival.

To determine whether oligodendrocyte damage in actively demyelinating MS lesions is also accompanied by myelinosome formation, we imaged myelin sheaths with higher resolution in brain sections immunostained for MBP and neurofilament (NF200). We detected focal myelin out-foldings that met the morphological criteria for myelinosomes as dynamically observed and ultrastructurally corroborated in the EAE model (Fig. 4f). Myelinosome density was elevated in the lesion area compared with the adjacent NAWM. No myelinosomes were present in the white matter of control brain sections from patients without neuroimmunological disease or in brain lesions of patients suffering from progressive multifocal leukoencephalopathy (PML), a disease, in which a viral attack results in oligodendrocyte death^22^ (Fig. 4g; Supplementary Fig. 6). Thus, also in MS, myelin damage corresponds to the presence of focal changes akin to myelinosomes, suggesting the occurrence of a local immune attack mediated by the abundant mononuclear phagocytes found in areas of active demyelination in MS (Fig. 4b,e).

## Discussion

Damage to oligodendrocytes and their myelin sheaths is the pathological hallmark of MS lesions. Here, we show that changes in oligodendrocyte morphology in neuroinflammatory lesions proceed from the outside-in—resulting in oligodendrocytes that have less and shorter processes. Remarkably, oligodendrocytes appear to be able to survive in this amputated state for extended periods of time, potentially preserving their ability to reform myelin sheaths. While the morphological consequence of a neuroinflammatory attack is thus the selective loss of the peripheral oligodendrocyte compartments, this does not exclude a predisposing global incapacitation of the affected oligodendrocytes, for example, due to an altered metabolic state or disrupted ion balance due to a toxic inflammatory milieu (a state of the oligodendrocyte that could be described as a ‘dying-back oligodendrogliopathy'^2^,^23^,^24^). Indeed the ability of therapeutic strategies that primarily target oligodendrocyte survival and function to also limit demyelination would argue for such a predisposing effect^25^,^26^. In the MS models and brain biopsies we investigated here, the centripetal pattern of oligodendrocyte damage we observed appears to be initiated at discrete sites on the surface of myelin sheaths, where in parallel to the progress of demyelination, local out-foldings—myelinosomes—are found. Electron microscopy analysis using high-pressure frozen samples to optimally preserve myelin morphology^27^,^28^ and serial sectioning electron microscopy for 3D reconstruction^14^,^15^ revealed that myelinosomes can be classified into several ultrastructural subtypes. In some cases, the entire myelin sheaths was detached from the underlying axon, while in other cases only the upper half of the myelin leaflets was lifted off, resulting in split myelin sheaths. Both of these patterns were sometimes accompanied by myelin inclusions. At least some of these phenomena could be explained by the recent insight that myelin basic protein (MBP), the major protein component of the myelin sheath, transitions from a soluble phase to a cohesive network to allow myelin compaction^29^. Such a protein phase transition could be locally reversed by pathological impact, allowing individual myelin leaflets to separate and collapse into myelin bulbs^30^.

Bulb-like myelin structures have indeed been noted in a number of situations, where myelin sheaths are attacked or remodelled, for example, in peripheral neuropathies such as Charcot–Marie–Tooth type 4H^31^, in animal models of dysmyelination^32^, as well as, already previously, in MS and its animal model^16^,^33^,^34^,^35^. Similar alterations also occur during development when excess myelin is formed, for example, in the optic nerve^27^. Still, when we analyzed models where the site of initial injury is expected to be at the oligodendrocyte soma (for example, ethidium bromide and cuprizone intoxication of oligodendrocytes, as well as a viral attack in human PML), we did not observe typical myelinosomes. This suggests that myelinosome formation is indicative of a centripetal progression of oligodendrocyte damage, possibly caused, or at least promoted, by stressors that attack the cell's periphery.

What might the local stressors be that induce myelinosome formation in neuroinflammatory lesions? Our analysis indicates that anti-myelin antibodies that bind and activate the complement cascade are important mediators of myelinosome formation. Such antibody–complement complexes could opsonize the myelin surface and make myelin a target for processes of bloodborne macrophages resulting in myelin stripping. This interpretation is in line with previous work implicating monocyte-derived macrophages as primary effectors of autoimmune demyelination^16^. In contrast, results obtained in the cuprizone model suggest that during toxic demyelination microglial cells rather than bone-marrow derived macrophages remove myelin^36^. Thus, there appears to be a mechanistic distinction between phagocytic clearance of myelin debris during repair after toxic oligodendrocyte injury^36^,^37^,^38^, and the inflammatory destabilization and stripping of myelin that mediates primary myelin damage in autoimmune disease models.

As antibody–complement complexes have also been detected in actively demyelinating MS lesions^4^,^39^, it is conceivable that the described cascade of myelin damage is also operational in human MS^40^. Indeed our analysis of actively demyelinating MS lesions showed that myelin was lost, while oligodendrocyte cell bodies were preserved. Notably, we focused our analysis on early active lesions, in which the presence of minor myelin constituents in the phagocyte cytoplasm indicates that demyelination has been initiated within the last 2–3 days^21^. While this time-frame should limit the contribution of cell replacement processes, it is difficult to exclude that some of the oligodendrocytes in the lesion centre and probably to a lesser extent at the lesion rim are recruited from local progenitor pools. Still our results are at least consistent with the view that in our samples of actively demyelinating MS lesions, which were classified as pattern I or pattern II lesions^41^, oligodendrocyte damage spreads in a centripetal pattern, with myelin as the first target of the immune attack. In other types of lesions, distinct mechanisms might well operate, as—for example—oligodendrocyte apoptosis is prominent in pattern III lesions^4^,^41^ and has also been described in particularly early, pre-phagocytic stages of actively demyelinating central nervous system (CNS) lesions^3^,^42^,^43^. In this context, it is interesting to note that lesions in PML, a disease in which extensive oligodendrocyte apoptosis is induced by the JC virus^22^, show no evidence of myelinosome formation. In any case, as pattern I and II lesions presumably represent a majority of actively demyelinating MS lesions^4^,^44^, our findings are relevant for many cases of MS. It was in such MS lesions, where we found a high density of myelinosomes comparable to that observed in acute EAE lesions. Interestingly, myelinosomes were present on normal-appearing axons in both EAE and MS lesions, indicating that myelin damage can be initiated independently of axon damage in neuroinflammatory lesions^6^ (Supplementary Fig. 7).

Taken together, our results suggest that the process of myelinosome formation is also present in actively demyelinating MS lesions, and that the presence of myelinosomes indicates the centripetal spread of morphological alterations in oligodendrocytes due to an immune attack on their periphery. Thus, interventions that target the cellular executors and molecular mediators of myelinosome formation might help to prevent myelin loss in MS.

## Methods

### Transgenic mice

C57BL/6 and Biozzi ABH mice were obtained from Janvier Labs and from Envigo, respectively. Reporter mice on a C57BL/6 background were crossed with Biozzi ABH mice to obtain (F1) *Thy1-YFP16*/Biozzi ABH mice, in which neurons were fluorescently labelled^12^; *PLP-GFP*/Biozzi ABH mice, in which the PLP promoter localizes the GFP specifically to oligodendrocytes^10^ and *CCR2*^*RFP/+*^*/*Biozzi ABH, in which circulating monocytes are labelled with RFP^16^. For the experiments, adult animals between 6 and 12 weeks of age from both sexes were used. All animal experiments were performed in accordance with regulations of the relevant animal welfare acts and protocols approved by the respective regulatory bodies (Regierung von Oberbayern).

### EAE induction

Active EAE was induced in adult mice by immunization with recombinant MOG or a synthetic MOG peptide. Biozzi ABH and Biozzi ABH/BL6 animals were immunized as described previously^7^ and received an emulsion containing 50 μg of purified recombinant MOG (N1-125) in complete Freund's adjuvant (containing 5–10 mg ml^−1^ *Mycobacterium tuberculosis* H37 Ra, Sigma-Aldrich) at day 0 and day 7. On day 0, 1, 7 and 8 following immunization, 50 ng of pertussis toxin was administered intraperitoneally. Mice on a C57BL/6 background were immunized with either 200 μg of MOG peptide (N35-55) in complete Freund's adjuvant as previously described^45^, followed by the injection of 400 ng of pertussis toxin at day 0 and day 2 after immunization; or 200 μg of recombinant rodent MOG (N1-125) in complete Freund's adjuvant, followed by the injection of 250 ng of pertussis toxin at day 0 and day 2 after immunization; or 100 μg of recombinant human MOG in complete Freund's adjuvant as previously described^46^, followed by the injection of 240 ng of pertussis toxin at day 0 and day 2 after immunization.

After immunization, mice were weighed daily and neurological deficits were evaluated according to the following EAE score: 0, no clinical signs; 0.5, partial tail weakness; 1, tail paralysis; 1.5, gait instability or impaired righting ability; 2, hind limb paresis; 2.5, hind limb paresis with dragging of one foot; 3, total hind limb paralysis; 3.5, hind limb paralysis and fore limb paresis; 4, hind limb and fore limb paralysis; 5, death.

### Ethidium bromide model

Chemical induction of demyelination was performed in *PLP-GFP*/Biozzi ABH mice as described previously^47^. Briefly, mice received a single injection of 0.5 μl of ethidium bromide (1 mg ml^−1^ ) into the ventral funiculus of the lumbar spinal cord, at a depth of 1.2 mm from the meningeal surface. Control animals were injected with 0.5 μl of saline using the same protocol. Mice were sacrificed 4 days after the injection.

### Immunohistochemistry

Animals were euthanized with isofluorane and perfused transcardially with 20 ml of saline solution followed by 20–30 ml of 4% paraformaldehyde (PFA) in 0.1 M phosphate buffer. Spinal cords were dissected out and post-fixed in 4% PFA overnight at 4 °C. To cryo-protect the tissue, it was incubated in 30% sucrose in 1 × PBS for 48 h before embedding the specimens in Tissue-Tek (O.C.T., Sakura Finetek Europe B.V.) compound and freezing it at −20 °C. Subsequently 20–30 μm thick longitudinal sections of the lumbar spinal cord were cut and stained free-floating. The sections were rinsed three times for 10 min in 1 × PBS at room temperature and then incubated for 1 h at room temperature with 10% goat serum. To stain myelin, a rabbit anti-MBP antibody was applied after cold methanol pre-treatment and to label phagocytes, a rabbit anti-Iba1 (antibody dilution 1:200, Wako) was used. The sections were first incubated overnight at 4 °C in a solution containing 1% goat serum/ 0.1% NaN_3_/ 0.5% Triton/ 1 × PBS in which the primary antibody was diluted. The sections were washed three times with 1 × PBS and incubated with a goat anti-rabbit Alexa 594 secondary antibody (dilution 1:1,000, Thermo Fisher Scientific) for 4 h at 4 °C in a solution containing 1% goat serum/ 0.5% Triton/ 1 × PBS. All samples were counterstained with the Nissl-like dye, Neurotrace 640/660 (1:500, Thermo Fisher Scientific). After staining, the samples were mounted with Vectashield and covered with a coverslip glass sealed with nail polish.

### Single-cell labelling

Healthy and immunized *PLP-GFP*/Biozzi ABH animals were used to study the morphology of single oligodendrocytes. Seven days after immunization (in case of EAE animals), mice were first anesthetized with ketamine and xylazine (ketamine 87 mg kg^−1^, Bremer Pharma GmbH; xylazine 13 mg kg^−1^, Serumwerk Bernburg AG) and the dorsal spinal cord was exposed as previously described^48^. Then 0.5 μl of rabies virus SAD ΔG mCherry was injected in the white matter of the lumbar spinal cord with a glass capillary at a depth of 0.2–0.4 mm. Each mouse received two injections. Animals were then killed 9 days after rabies virus injections. For two-photon time-lapse experiments, mice were imaged at weight loss, at the onset of clinical symptoms, and at 1 or 2 days after the onset, and killed after the imaging session (6–9 days after rabies virus injection).

Quantifications of the process number and length of single oligodendrocytes were performed manually based on 3D-rendered confocal images stacks (rendering was performed with Imaris Scientific 3D/4D Image processing software). The total number of primary processes exiting the soma was counted and expressed as the average number of processes per OL; the length of all the processes (primary and other) was measured and divided by the number of processes to obtain average process length. Images from both healthy and EAE groups were analyzed by an evaluator blinded to the group status of the images.

### Histopathology

For histopathological analysis Biozzi ABH, Biozzi ABH/BL6 and *Thy1-YFP16*/BL6 mice were transcardially perfused with 4% PFA and paraffin-embedded according to standardized protocols. Stainings were done on 3–4 μm thick cross-sections of lumbar, thoracic and cervical spinal cords. LFB/PAS-staining was performed to assess demyelination, and the demyelinated area of spinal cross-sections (expressed as % of total white matter) was measured manually using Pannoramic Viewer 1.15 software (http://www.3dhistech.com/pannoramic_viewer). Images from both healthy and EAE groups were analyzed by an evaluator blinded to the group status of the images.

To assess axonal pathology, sections were stained with Bielschowsky silver impregnation. For automatic quantification of axon density a custom ruleset was created using Definiens Developer software (Definiens). First, regions of interest were exported from 400 × slide scans in full resolution using Pannoramic viewer. On these, tissue-background separation was performed and the total area of tissue calculated. Axons were detected and nuclei excluded based on their respective characteristic spectral properties (for example, brightness, RGB channel distribution) and morphology (for example, surrounded by cytoplasm). The number of axons was quantified within a lesion and in the respective perilesion area (considered as normal-appearing white matter). Axon numbers were first exported as axon density [#mm^−2^], and subsequently expressed in % normalized to the NAWM.

Immunostainings were carried out after antigen retrieval with microwave pre-treatment (citrate buffer, pH 6). Non-specific antibody binding was inhibited with 10% FCS in PBS. Immunostaining was done using following primary antibodies: rat anti-Mac3 (macrophages/activated microglia, 1:200, BD), T cells with an anti-CD3 (T cells, 1:50, Serotec). Bound primary antibodies were visualized by an avidin-biotin technique with 3,3′-diaminobenzidine 3 (DAB) as chromogen (haemalaun counterstaining of nuclei). Stained tissue sections were scanned at × 200 magnification using a panoramic 250 flash slide scanner (3D Histech Ltd). For the quantification of immune cell density, images were opened with the software Pannoramic Viewer and exported as TIFF-files to ImageJ. To count the number of Mac3+ cells and the number of CD3+ cells, the ‘cell counter' plugin was used.

### Human tissue analysis

For histopathological analysis, brain sections derived from biopsies of MS lesions (classified as early active pattern I or II lesions as described previously^4^; for details see Supplementary Table 1), from autopsy cases of PML or from control white matter derived from autopsy cases without neuroimmunological diseases were fixed in 4% PFA and embedded in paraffin according to standardized protocols. Their use for scientific purposes was in accordance with institutional ethical guidelines and approved by the ethics committee of the University of Göttingen (Germany). Informed consent was obtained from all subjects. Immunostainings were carried out on 2–3 μm thick sections after antigen retrieval with microwave pre-treatment (citrate buffer, pH 6). Non-specific antibody binding was inhibited with 10% FCS in PBS. The following primary antibodies were used: rabbit anti-myelin basic protein (MBP; 1:1,000, Dako), mouse anti-NogoA (oligodendrocytes, 1:10,000, mAb 11C7, a generous gift from M.E. Schwab, Brain Research Institute, ETH and University of Zürich) and Alexa647 pre-labelled rabbit anti-Iba1 (microglia/macrophages; 1:1,000, WAKO, antibody labelling was done with Alexa Fluo 647 antibody labelling kit, Molecular Probes) and anti-Neurofilament 200 (axons, 1:50, Dako). Bound primary antibodies were visualized using appropriate species-specific Alexa488- or Alexa555-conjugated secondary antibodies (all from Jackson ImmunoResearch Laboratories Inc) and counterstained with Neurotrace 435 (Thermo Fisher Scientific) or DAPI. Stained tissue sections were scanned at × 400 magnification using a panoramic 250 flash slide scanner.

### Confocal microscopy

Samples were scanned on an upright FV1000 confocal microscopy system (Olympus) equipped with × 10/0.4 air, × 20/0.85 and × 60/1.42 oil immersion objectives. Images were acquired using standard filter sets and processed with ImageJ or Adobe Photoshop software.

### Molecular manipulation of demyelination

To assess the contribution of antibodies and the complement system to myelinosome formation we used *Thy1-YFP16*/BL6 mice. To induce primary demyelination the anti-MOG monoclonal antibody 8.18C5 (purified hybridoma supernatant with 35 μg of mouse IgG per injection, kindly provided by Dr. Krishnamoorthy, Max-Planck Institute of Biochemistry) was injected intraperitoneally for three days starting from disease onset^18^. An IgG1 isotype control antibody (100 μg per injection) was injected at similar time-points in the control group. To deplete complement 25 μg of Cobra Venom Factor^49^ (1 μg μl^−1^, Quidel Corporation) was injected at the day of disease onset followed by three injections with anti-MOG monoclonal antibody 8.18C5 (at Onset, Onset+1, Onset+2). All mice were weighed and scored daily (as described above) and sacrificed three days after onset of clinical signs.

### *In vivo* imaging

After anaesthesia with ketamine and xylazine (ketamine 87 mg kg^−1^, xylazine 13 mg kg^−1^) or with a combination of medetomidin (0.5 mg kg^−1^, Orion Pharma), midazolam (5 mg kg^−1^, Ratiopharm) and fentanyl (0.05 mg kg^−1^, B. Braun Melsungen AG) animals were placed on a heating pad for 15 min. Tracheotomy and intubation were performed to minimize breathing artefacts by controlling respiration during imaging and the dorsal spinal cord was surgically exposed as previously described^7^,^13^,^48^; the opening was constantly superfused with artificial cerebrospinal fluid (aCSF; prepared according to the protocol provided by Alzet, http://www.alzet.com/products/guide_to_use/csf_preparation.html). A spinal clamping device (spinal adaptor; Narishige STS-A) was used to stabilize the animal and an agarose well (4% agarose) surrounding the spinal opening was formed to keep the spinal cord covered with aCSF and to incubate with vital dyes as previously described^13^. For *in vivo* imaging, we used the following two-photon microscopes: a custom-built setup based on an Olympus FV300 and a commercially available Olympus FV1200 MPE. Both microscopes were equipped with femto-second pulsed Ti:Sapphire lasers, which were attenuated by a polarization-based beam splitter or acousto-optical modulators, respectively.

To study myelin damage *in vivo*, immunized *Thy1-YFP16*/Biozzi ABH mice with an EAE score ≥2.5 were imaged. After bath application of MitoTracker Red (Thermo Fischer Scientific) at a concentration of 8 μM for 30 min (an application scheme that primarily reveals myelin^13^) the spinal cord was carefully washed with aCSF before the imaging session. The laser was tuned to 910 nm to excite both YFP (axons) and MitoTracker Red (myelin) and fluorescence emission was detected using a green/red filter set (BA495-540, BA575-630). The presence of damaged axons, myelin debris and a high density of nuclei, labelled by the nuclear dye POPO-1 Iodide (Thermo Fischer Scientific) as previously described^13^, were used to identify inflamed areas of the dorsal white matter where imaging was performed. To monitor changes in myelin morphology over time, image stacks were acquired every 10 min for several hours. Time-lapse images were acquired with a × 25/1.25 water immersion objective with a pixel size of 0.082 μm pixel^−1^ and a pixel dwell time of 2 μs.

To study oligodendrocyte damage at the single-cell level, *PLP-GFP*/Biozzi ABH mice were immunized and injected with SADΔG-mCherry rabies virus (as described above). Mice were imaged at weight loss, at the onset of clinical symptoms or one and two days later. The laser was tuned to 840 nm to excite GFP (oligodendrocytes) and to 910 nm to visualize the virus-induced expression of mCherry. Single oligodendrocytes were imaged for 270–330 min; time-lapse images were obtained with a × 25/1.25 water immersion objective and a pixel dwell time of 2 μs.

Similar imaging experiments were performed in healthy animals with or without SADΔG-mCherry rabies virus injection, to make sure that the morphological changes in myelin structure and oligodendrocyte processes were not due to anaesthesia, surgery, photo-, dye-or virus-toxicity. In control mice neither signs of myelin alterations (*n*=3 mice each imaged for 2–3 h) nor other signs of oligodendrocyte damage (*n*=5 mice each imaged for 270–300 min) were detected even after several hours of imaging.

### Tissue fixation for correlated light and electron microscopy

*Thy1-YFP16*/Biozzi ABH animals with an EAE score of ≥2.5 were transcardially perfused with 2.5% electron microscopy grade glutaraldehyde (GA) and 2% PFA in 0.1 M phosphate buffer (pH 7.2). The spinal cords were isolated and post-fixed in 2.5% GA/ 2% PFA overnight. The tissue was then embedded in 4% agarose and cut into thick sections (100–150 μm) using a vibratome. To visualize myelin, MitoTracker Red (at a concentration of 8 μM) was applied for 5 h at room temperature. Then sections were carefully washed three times for 15 min in 1 × PBS and mounted in 1 × PBS under a coverslip in imaging chambers built with parafilm spacers on a glass slide^15^.

### NIRB and electron microscopy

MitoTracker Red-labelled samples were imaged on an Olympus FV1200 MPE using confocal laser excitation at 515 and 559 nm (ExDM405/488/559/635, BA505-540, BA575-620) to detect myelinosomes. Once the myelin out-folding was located, NIRB marks were placed using the Ti:Sapphire laser tuned to 910 nm and a pixel dwell time of 10 μs pixel^−1^ with a × 25/1.05 water objective^15^. To facilitate the detection of the structure of interest a bigger box of 100 × 100 μm was burnt 2 μm above the myelinosome. The serial sectioning of the tissue containing the NIRB marks was performed as described previously^15^. Electron micrographs were obtained at 120 KV using a JEOL JEM-1400 equipped with a Gatan Ultrascan 1000XP camera. Images were then processed with the software Reconstruct (http://synapses.clm.utexas.edu/tools/reconstruct/reconstruct.stm), while for rendering we used 3DS Max software (Autodesk).

### Preparation and microscopy of high-pressure frozen samples

MOG-immunized Biozzi AbH mice and mice fed with 0.2% of the copper chelator cuprizone, were killed during the progressive phase of EAE (about 25–30 days after immunization) or 2 or 3 weeks after cuprizone feeding, respectively, by cervical dislocation and the spinal cord was quickly dissected. After embedding in 10% gelatine (Twee Torens), 200 μm thick cross-sections of the spinal cord were cut with the help of the Leica vibratome VT1200S. The sections were assessed macroscopically and lesion-containing regions were cryofixed in 20% poly(vinyl-pyrrolidinone) (Sigma-Aldrich) using the high-pressure freezer Leica HPM100. The tissue samples were then freeze-substituted using the ‘tannic acid and OsO_4_'-protocol in a semi-automated fashion on the Leica AFS II as described previously^27^,^28^. After Epon embedding of the tissue, the samples were cut with a Leica Ultracut S ultramicrotome into 0.5 μm semithin sections. These cross-sections were subsequently stained with methylene blue/Azure II blue for 1 min to visualize myelin (lipid-rich) areas in the tissue. Areas devoid of this staining were considered regions of interest that contain lesions sites. Ultrathin sections of 50 nm thickness of these areas were cut thereafter and contrasted with 4% uranylacetate for 20 min at room temperature^28^ (SPI-Chem). Electron micrographs were obtained on a LEO EM912AB electron microscope (Zeiss) equipped with an on-axis 2k CCD-camera (TRS) using the ITEM (Olympus) software.

### Image processing and analysis

To analyze the spread of oligodendrocyte damage, *PLP-GFP*/Biozzi ABH mice were immunized and perfused with 4% PFA at weight loss, onset of clinical symptoms, two days after onset, and 20 days after the second bout of EAE. Longitudinal sections of the lumbar spinal cord were cut and stained free-floating with an anti-MBP antibody and NeuroTrace 640/660 as described above. For each animal, we analyzed three lesions (each lesion was separated into the following regions: lesion centre, lesion border and adjacent normal-appearing white matter). ImageJ was used for processing and quantification of the areas. The ‘cell counter' plugin was used to count the number of oligodendrocyte cell bodies and the number of all nuclei. The length of myelin was traced using the ‘freehand line' tracing tool and the ‘measure' plugin, and expressed in mm. The quantification of myelinosome density in *Thy1-YFP16*/Biozzi ABH at different time-points of the disease course, in *Thy1-YFP16*/BL6 mice treated with 8.18C5/ CVF+8.18C5 or isotype control antibody, mice immunized with recombinant MOG or human MOG, and mice injected with ethidium bromide, as well as in human samples (MS, control and PML cases), was performed as follows. Sections were stained with anti-MBP as described above. High magnification confocal images were taken with a × 60/1.42 oil objective (with × 4 zoom at 0.082 μm pixel^−1^) at the border of the lesions. Quantification was performed using ImageJ and its ‘trace' and ‘measure' plugins: the length of each axon in the stack was measured and the density of myelinosomes was expressed as number of myelinosomes per total mm of axons analyzed. Myelinosomes were defined as bulb-like myelin out-foldings that were still connected to the main myelin sheath. For the analysis of the human samples, images were opened with the software Pannoramic Viewer (http://www.3dhistech.com/pannoramic_viewer) and exported as TIFF-files to ImageJ. To count the number of Iba1+ cells and the number of NogoA+ cells, the ‘cell counter' plugin was used. The length of myelin was measured with the ‘trace' and ‘measure' plugins, as described before. The density of myelinosomes was measured as described above for the murine model.

### Statistical analysis

Results are given as mean±s.e.m. Data sets were tested for normal distribution using the D'Agostino–Pearson test. Those data sets for which a normal distribution could be assumed were then compared using student *t*-test or, if there were more than two samples, analyzed using one-way ANOVA followed by Bonferroni's multiple comparison tests. When normal distribution could not be assumed, the nonparametric Mann–Whitney test or, if there were more than two samples, the Kruskal–Wallis followed by Dunn's multiple comparison tests were chosen. Statistical analyses were performed with GraphPad Prism software.

### Data availability

All data generated or analyzed during this study are included in the published article (and its Supplementary Information file) or are available from the corresponding authors upon reasonable request.

## Additional information

**How to cite this article:** Romanelli, E. *et al*. Myelinosome formation represents an early stage of oligodendrocyte damage in multiple sclerosis and its animal model. *Nat. Commun.* **7,** 13275 doi: 10.1038/ncomms13275 (2016).

**Publisher's note:** Springer Nature remains neutral with regard to jurisdictional claims in published maps and institutional affiliations.

## Supplementary Material

Supplementary Information Supplementary Figures 1-7 and Supplementary Table 1

Supplementary Movie 1 Formation of a myelinosome in acute neuroinflammatory lesion. *In vivo* multi-photon time-lapse of myelinosome formation in the lumbar spinal cord of a Thy1-YFP16/BiozziABH mouse imaged two days after EAE onset. Axon, white; myelin, magenta.

Supplementary Movie 2 Three-dimensional ultrastructure of myelinosomes. 3D-rendering of two myelinosomes based on serial sectioning electron microscopy. Two adjacent immune cells are contacting the myelinosomes and engulfing them with their processes. Axon, white; myelin, magenta; immune cells, blue and cyan.
