# Supplementary material for: Myelinosome formation represents an early stage of oligodendrocyte damage in multiple sclerosis and its animal model
Source: Nat Commun. 2016 Nov 16;7:13275. doi: 10.1038/ncomms13275 (PMC5116090; doi:10.1038/ncomms13275)
Supplement: Supplementary Information — Supplementary Figures 1-7 and Supplementary Table 1 [file ncomms13275-s1.pdf]

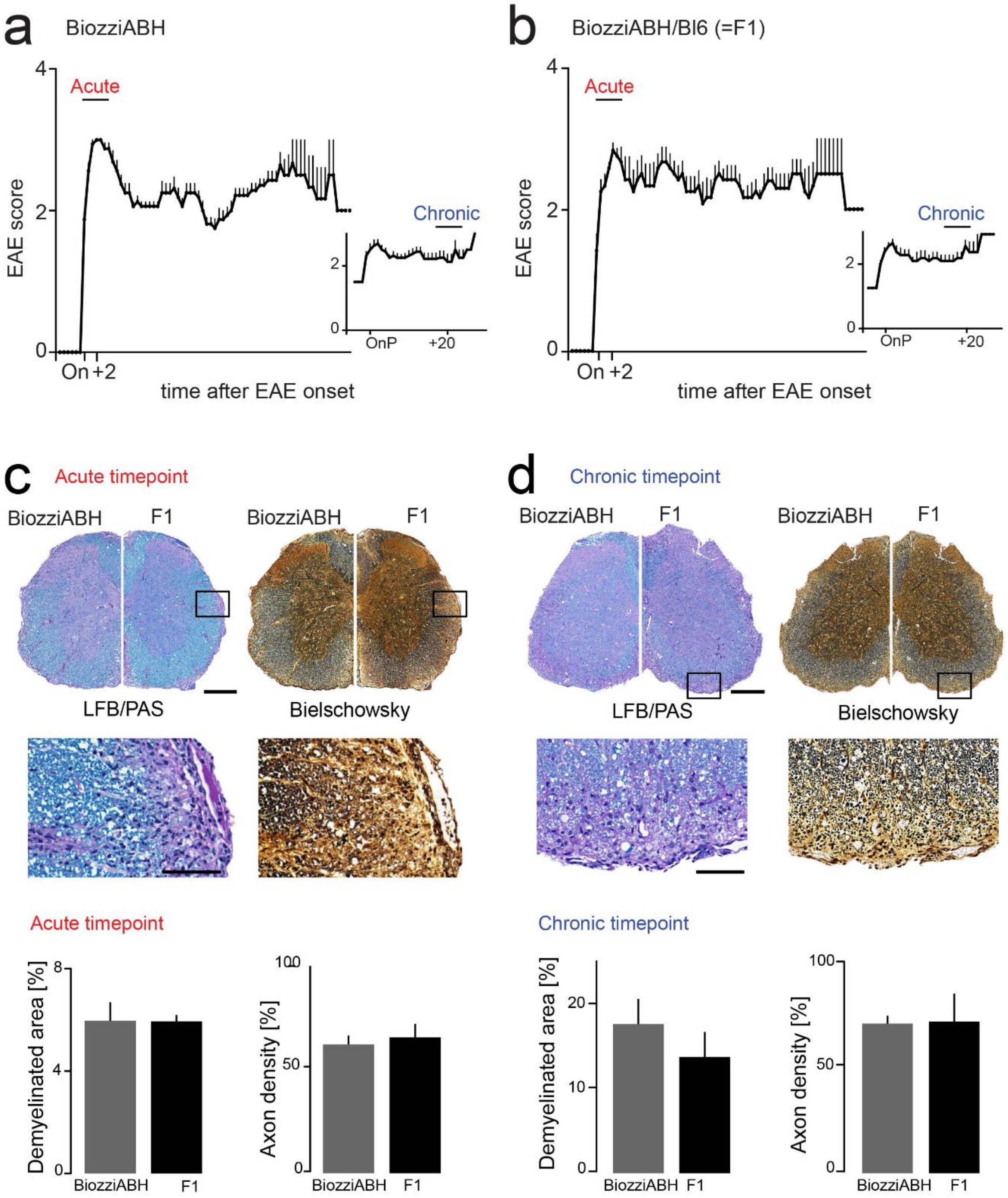
a
BiozziABH
Acute
Chronic
EAE score
OnP
+20
On +2
time after EAE onset
b
BiozziABH/Bl6 (=F1)
Acute
Chronic
EAE score
OnP
+20
On +2
time after EAE onset
c
Acute timepoint
BiozziABH
F1
BiozziABH
F1
LFB/PAS
Bielschowsky
d
Chronic timepoint
BiozziABH
F1
BiozziABH
F1
LFB/PAS
Bielschowsky
Acute timepoint
Demyelinated area [%]
BiozziABH
F1
Axon density [%]
BiozziABH
F1
Chronic timepoint
Demyelinated area [%]
BiozziABH
F1
Axon density [%]
BiozziABH
F1

Supplementary Figure 1 - F1 offspring of BiozziABH mice show unchanged acute and chronic EAE. (**a**,**b**) Clinical course of EAE in pure BiozziABH (**a**) and BiozziABH/Bl6 F1 (**b**) is comparable (n=6-8 mice for each genotype). (**c-d**) Histopathology of BiozziABH and F1 animals is comparable at acute (**c**) and chronic (**d**) time-points of the disease as evidenced by overview images (top panels), high power micrographs (middle, boxed areas in top panels) and quantitative analysis (bottom, n=5 mice for each genotype) of LFB/PAS (to assess demyelination) and Bielschowsky stainings (to assess axon density). Scale bar in **c** and **d**, 200 µm (overview) and 50 µm (inset). No significant changes between the groups were detected (Mann-Whitney test; **acute timepoint**: demyelinated area, P = 0.6905; axon density, P = 0.8413; **chronic timepoint**: demyelinated area, P = 0.5476; axon density, P = 0.3095).

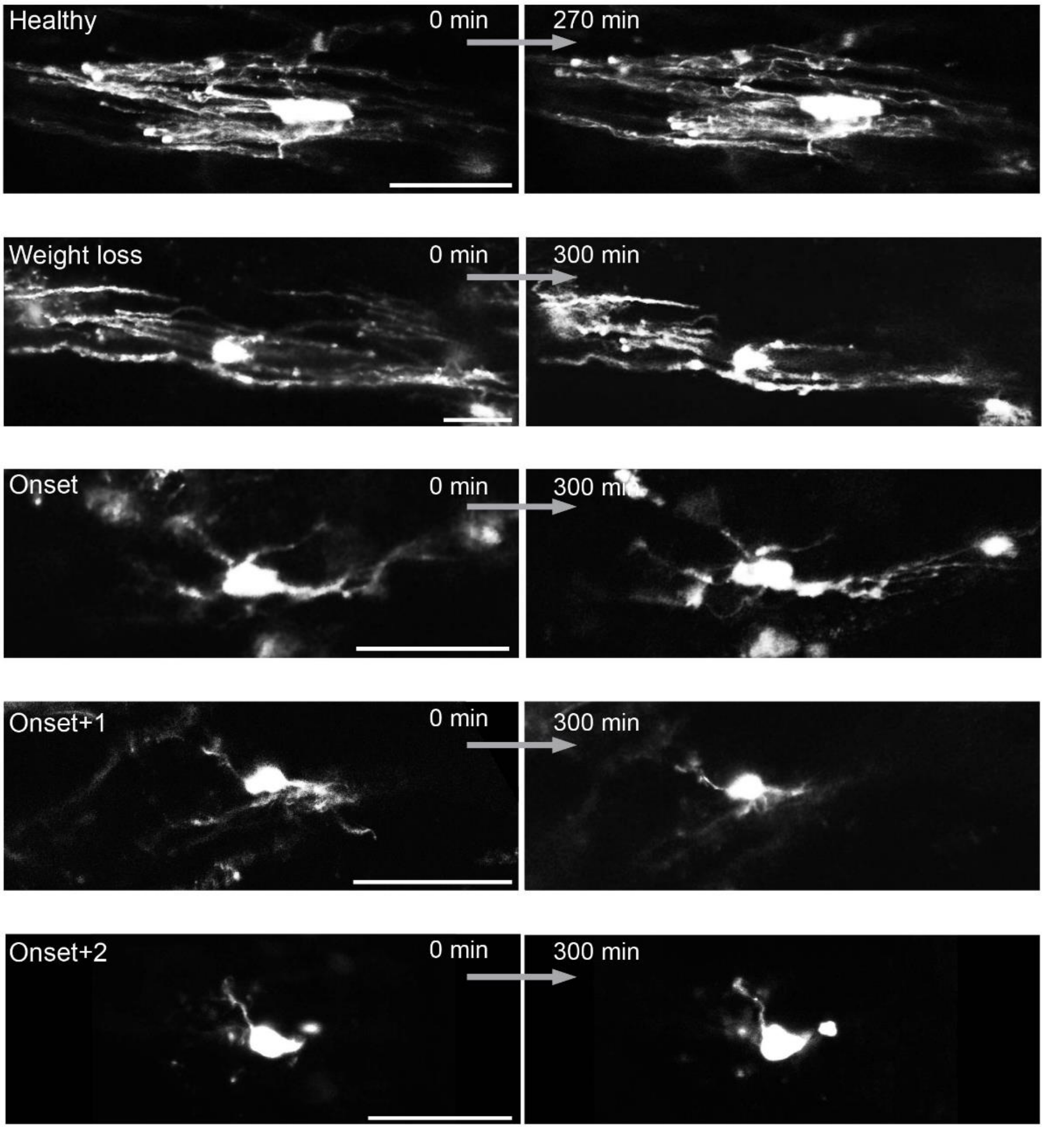

Supplementary Figure 2 – In vivo progression of oligodendrocyte damage in neuroinflammatory lesions. Single oligodendrocytes were imaged for up to 5 hours in healthy mice and at different time points of the EAE time course (Weight loss, Onset, Onset+1 and Onset+2). Time lapse imaging shows that oligodendrocytes can survive in an amputated state for several hours. Scale bars, 50 µm.

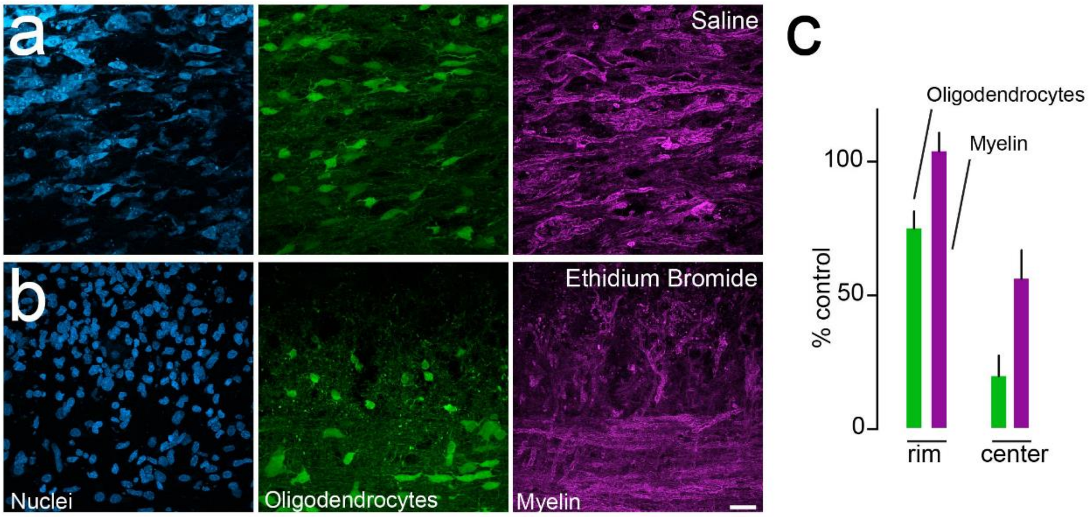

Supplementary Figure 3 – Oligodendrocyte damage induced by ethidium bromide shows "centrifugal" pattern. (**a,b**) Confocal images of spinal cord sections of *PLP-GFP*/BiozziABH mice injected with saline (**a**) or ethidium bromide (**b**) (oligodendrocytes are labeled with GFP, green; nuclei with NT, cyan; myelin with MBP, magenta). (**c**) Quantification of oligodendrocyte numbers (green bars) and myelin lengths (magenta bars) are shown as percentage of the control (n=3 mice for saline injection, n=3 mice for ethidium bromide injection). Scale bar in **a** (also for **b)**, 25 µm.

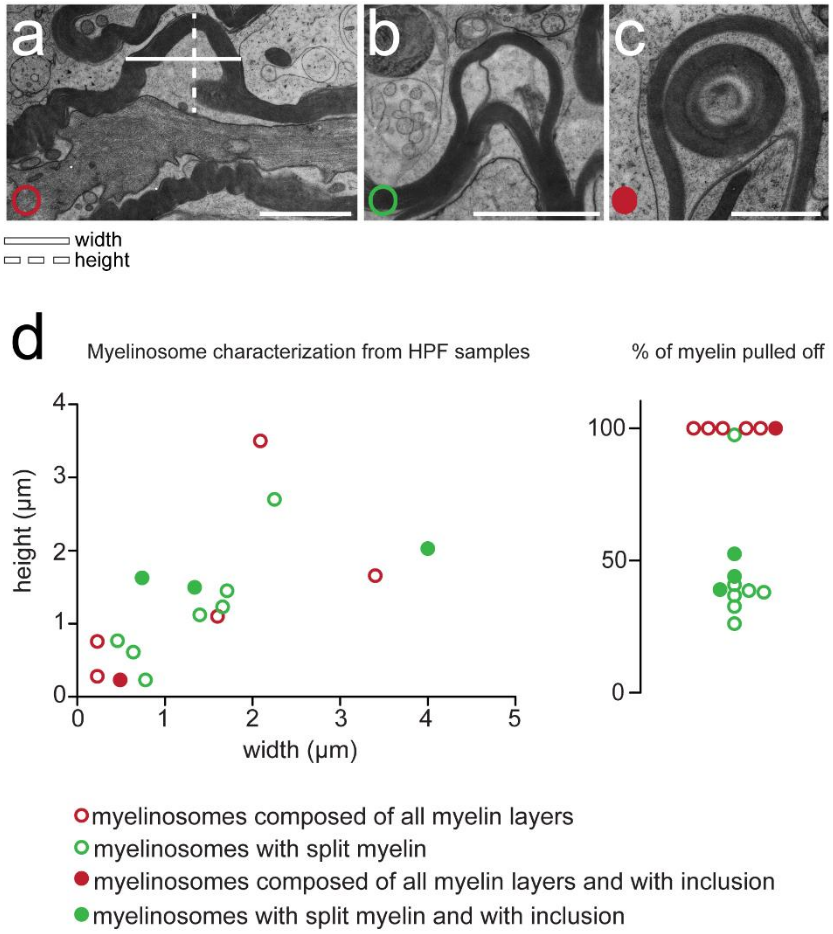

Supplementary Figure 4 – Ultrastructural characterization of myelinosomes in high-pressure frozen samples. (**a-c**) Electron micrographs from high pressure frozen spinal cord of EAE mice illustrating the presence of different types of myelinosomes (re-plot from **Fig. 2** without pseudo-coloring of myelin; myelinosome type is indicated by different symbols defined below panel **d**). (**d**) Quantitative characterization of myelinosome ultrastructure showing the width and height of individual myelinosomes (left panel), and the percentage of myelin that is detached from the axonal membrane (right panel) in relationship to the myelinosome type (indicated by different symbols defined below panel **d**; n = 16 myelinosomes analysed). Scale bar in **a**, 2 µm; **b**, 2 µm; **c**, 1 µm.

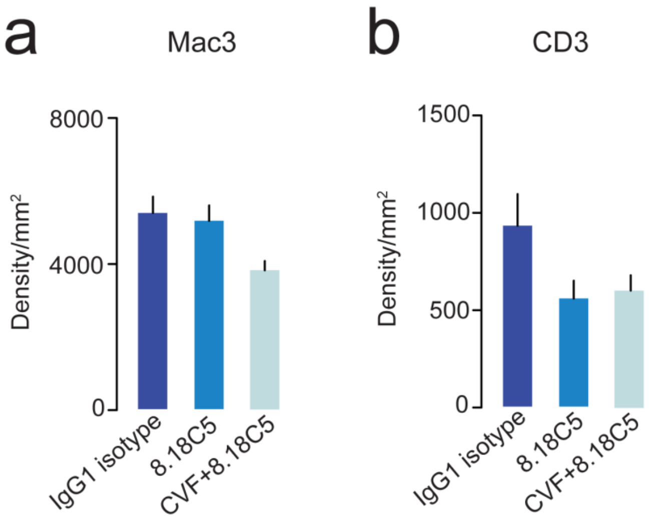

Supplementary Figure 5 - Anti-myelin antibody transfer and complement depletion does not exacerbate cellular infiltration in EAE. (**a-b**) Neither densities of macrophages/microglia (**a**), nor of T lymphocytes (**b**) are increased in EAE lesions after anti-MOG antibody ("8.18C5") treatment and complement depletion ("CVF"; n=11 mice for control, "IgG1 isotype"; n=10 mice for 8.18C5; n=4 mice for CVF+8.18C5). No significant differences between the groups were detected (Kruskal-Wallis followed by Dunn´s multiple comparison tests).

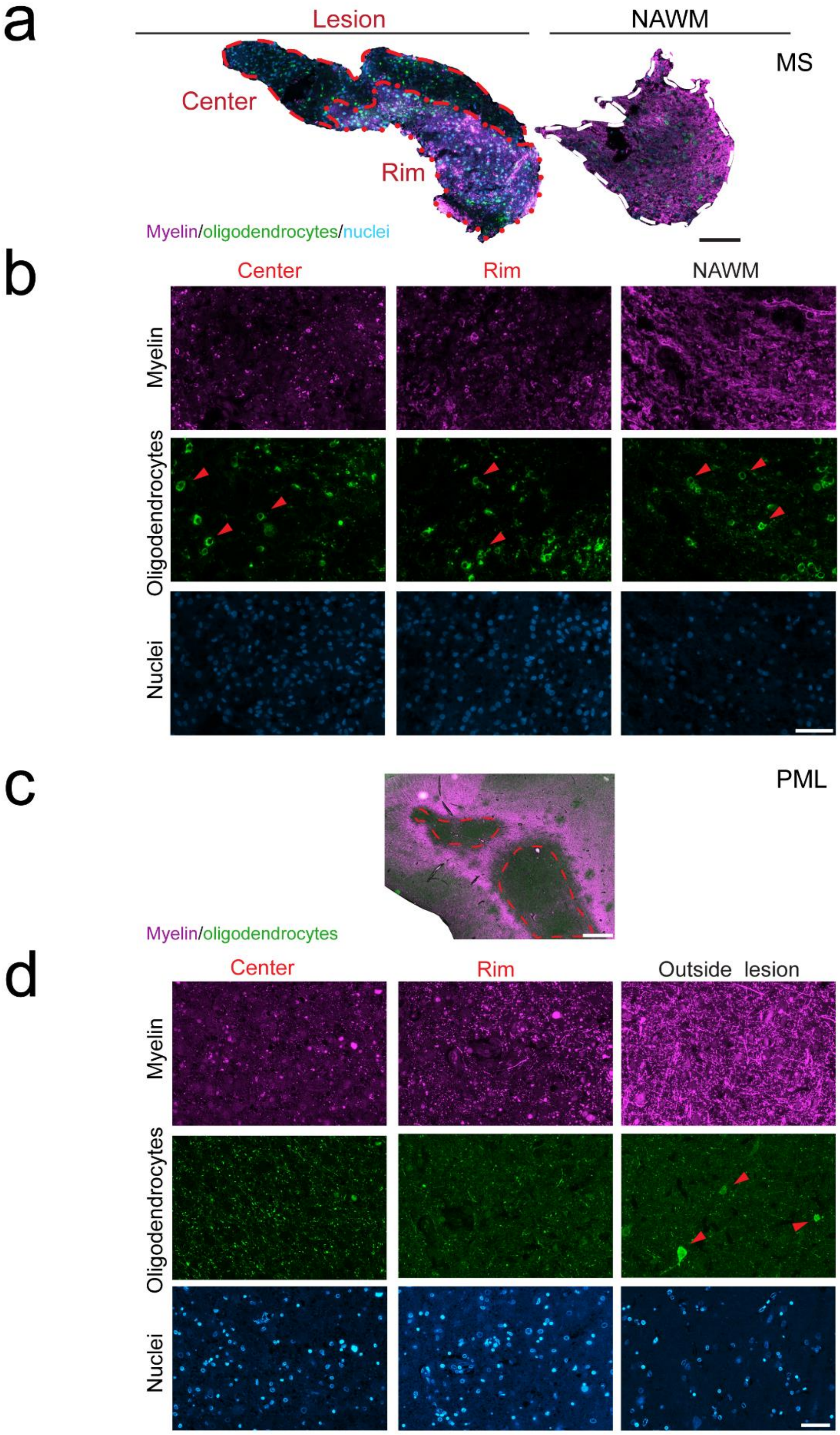
a
Lesion
NAWM
MS
Center
Rim
Myelin/oligodendrocytes/nuclei
b
Center
Rim
NAWM
Myelin
Oligodendrocytes
Nuclei
c
PML
Myelin/oligodendrocytes
d
Center
Rim
Outside lesion
Myelin
Oligodendrocytes
Nuclei

Supplementary Figure 6 – Oligodendrocyte pathology in progressive multifocal leukoencephalopathy and in actively demyelinating MS lesions. (**a**) Overview of two MS biopsies showing an actively demyelinating MS lesion and its rim as well as NAWM**.** (**b**) Higher magnification views from areas as defined in **a** (stained for myelin, magenta and oligodendrocytes, green, while nuclei were counterstained with DAPI, cyan). Arrowheads, oligodendrocyte somata. (**c**) Overview of a PML autopsy stained for oligodendrocytes (NogoA, green) and myelin (MBP, magenta); two large lesions are highlighted by a dashed line. (**d**) Higher magnification views from the center and rim of a lesion, as well as from the adjacent area outside the lesion (stained for myelin, magenta, and oligodendrocytes, green, while nuclei were counterstained with DAPI, cyan). Arrowheads, oligodendrocyte somata. Scale bar in **a**, 100 µm; **b**, 40 µm; **c**, 1 mm; **d**, 50 µm.

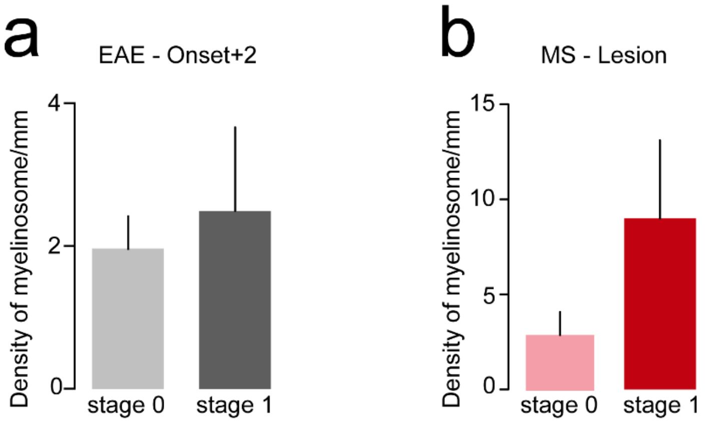

Supplementary Figure 7 – Relation of myelinosome formation and axon pathology in MS and EAE. The graphs show the density of myelinosomes in normal appearing (stage 0) and swollen (stage 1) axons in EAE lesions 2 days after onset of clinical symptoms (**a,** n=6 mice analysed) and in actively demyelinating MS lesions (**b**, n=7 biopsies analysed). Both in EAE and in MS no significance differences in the myelinosome density were detected between normal appearing and swollen axons (Mann-Whitney test; **a**, P = 0.9372; **b**, P = 0.2958).

Supplementary Table 1 – Clinical data of MS biopsies.

MS biopsies	1	2	3	4	5	6	7	8	9	10	11	12
Lesional activity	EA	EA	EA	EA	EA	EA	EA	EA	EA	EA	EA	EA
Lesion pattern	II	II	II	I	II	II	II	II	II	II	I	II
Gender	female	male	female	male	female	male	female	female	female	female	female	female
Age at biopsy	61.04	28.22	52.69	n.a.	35.66	30.89	54.69	73.17	34.31	29.00	67.16	20.25
Disease duration in years	0.04	10.23	12.47	n.a.	2.89	0.06	8.99	0.43	0.15	0.07	0.10	0.08
Time interval*	0.04	0.07	0.06	n.a.	0.15	0.06	0.04	0.43	0.15	0.07	0.10	0.08
MS disease course at biopsy	MP	SPMS	RRMS	MP #	RRMS	MP	RRMS	PPMS	MP	MP	MP	MP

* Time interval from first symptoms of index lesion to biopsy in years
# Presumed

EA: Early active
MP: Monophasic
SPMS: Secondary Progressive MS
RRMS: Relapsing-Remitting MS
